# Analysis of the Effect of Degree Correlation on the Size of Minimum Dominating Sets in Complex Networks

**DOI:** 10.1371/journal.pone.0157868

**Published:** 2016-06-21

**Authors:** Kazuhiro Takemoto, Tatsuya Akutsu

**Affiliations:** 1 Department of Bioscience and Bioinformatics, Kyushu Institute of Technology, Iizuka, Fukuoka, Japan; 2 Bioinformatics Center, Institute for Chemical Research, Kyoto University, Uji, Kyoto, Japan; University of Bristol, UNITED KINGDOM

## Abstract

Network controllability is an important topic in wide-ranging research fields. However, the relationship between controllability and network structure is poorly understood, although degree heterogeneity is known to determine the controllability. We focus on the size of a minimum dominating set (MDS), a measure of network controllability, and investigate the effect of degree-degree correlation, which is universally observed in real-world networks, on the size of an MDS. We show that disassortativity or negative degree-degree correlation reduces the size of an MDS using analytical treatments and numerical simulation, whereas positive correlations hardly affect the size of an MDS. This result suggests that disassortativity enhances network controllability. Furthermore, apart from the controllability issue, the developed techniques provide new ways of analyzing complex networks with degree-degree correlations.

## Introduction

Controlling complex systems is a central challenge in a wide range of research fields (e.g., molecular networks and brains in biology, the Internet and WiFi communication in telecommunications engineering, and economic science). Since complex systems are often represented as networks, a network perspective is helpful for understanding the control and design of the systems [1, 2], especially on networks with a scale-free property (i.e., a power-law distribution of degree *k*: *P*(*k*) ∝ *k*^−*γ*^) [1].

Liu et al. [3] proposed network controllability characterized by the minimum set of driver nodes (so-called *structural controllability*), which is sufficient to control the whole-system dynamics and is identified based on the maximum matching of the network. They found that network controllability is mainly determined by degree distributions; in particular, they showed that real-world networks, which are generally sparse and heterogeneous, are difficult to control.

It is also important to consider assortativity or degree-degree correlation [4], a structural property widely observed in real-world networks, when discussing the relationship between network structure and system dynamics because assortativity influences the system dynamics. For example, a negative degree-degree correlation (i.e., disassortativity) protects networks against the spread of viruses [5]. Disassortativity enhances synchronization [6], and it promotes observability [7]. Degree-degree correlation indicates the relationship between the node degrees, and it is often defined as the Pearson correlation coefficient of degrees between a connected node pair [4] (i.e., assortative coefficient *r*_*a*_). In particular, a negative and a positive *r*_*a*_ indicate the disassortativity and assortativity of networks, respectively. Pósfai et al. [8] investigated the effect of degree-degree correlation on network controllability in the context of driver nodes or maximum matching, and they showed that positive and negative degree-degree correlations tend to increase the number of driver nodes (i.e., they reduce network controllability); however, degree correlation coefficients have quadratic or no relationship with the number of driver nodes, depending on the nature of the underlying correlations.

However, more careful examinations are required to conclude the effect of degree-degree correlation because network controllability is also investigated in the context of minimum dominating sets (MDSs) [9–16] whose approach is conceptually similar to the controlling link dynamics [17]. In this context, network controllability is defined as the proportion of the MDS to network size. In particular, heterogeneous networks are known to show relatively small MDS sizes (i.e., they are easy to control) [10, 11]; however, this result does not conflict with the conclusion obtained by Liu et al. [3] because the MDS-based approach employs a stronger assumption that each node in an MDS can control its outgoing edges independently, although it also considers structural controllability under linear dynamics. Apart from the traditional control theory based on continuous/discrete dynamics, the MDS is important in the control of discrete engineering systems. The MDS is a well-known concept in graph theory and has already been applied to the design and/or control of various types of discrete engineering systems, which include mobile ad hoc networks (MANET) [18–20], transportation routing [21], and computer communication networks [21, 22].

Molnár et al. studied the relation between degree-degree correlation and MDS size [13]. They employed a probabilistic approach developed by Alon and Spencer and analyzed the expected size of a random dominating set (RDS) and a cutoff dominating set (CDS) under degree-degree correlations, where RDS is a dominating set (DS) obtained by randomly selecting nodes with some specified probability and adding the nodes not dominated by these nodes, and CDS is a dominating set obtained by selecting nodes above a degree threshold and adding the nodes not dominated by these nodes (see Ref [13] for details). Note that RDS and CDS provide the upper bounds of the size of an MDS (i.e., they are dominating sets but are not necessarily the minimum ones), with CDS giving better estimates in general than RDS does. Although their analytical methods provide accurate estimates of the size of an RDS and a CDS, they also showed experimentally that there is a large gap between the sizes of an MDS and an RDS (respectively, a CDS). Therefore, an accurate estimate of the size of an MDS under degree-degree correlation remains an unsolved problem.

In this study, therefore, we investigated the impact of degree-degree correlation on the size of an MDS both analytically and numerically, and showed that disassortativity, which indicates that high-degree nodes tend to connect to low-degree nodes, reduces the size of an MDS. To fill the known gap between the sizes of an MDS and an RDS, we developed two novel techniques: (i) decomposition of assortative (respectively, disassortative) networks, (ii) use of recursive probabilistic estimation methods for analyzing the size of an MDS in *k*-regular random graphs and (*h*, *k*)-regular random bipartite graphs. The former one is based on the proper understanding of assortative and disassortative network structures. The latter one is considered as a recursive application of the analysis technique used in Ref [13]. Although we applied both of them to the analysis of the size of an MDS, they are general and thus might become useful theoretical tools for analyzing various properties of assortative or disassortative networks.

## Methods

We used network models to show the validity of the theoretical results and to perform numerical simulations. In particular, the Chung—Lu (CL) model [23] was used to generate undirected scale-free (SF) networks with *N* nodes and *L* edges (i.e., average degree 〈*k*〉 = 2*L*/*N*). In the CL model, edges are drawn between nodes randomly selected according to node weight (*i* + *i*_0_ − 1)^*ξ*^, where *ξ* ∈ [0, 1) and *i* denote the node index (i.e., *i* = 1, …, *N*), where the constant *i*_0_ is considered to eliminate the finite-size effects (assortativity, in particular) [24]. A generated network shows that *P*(*k*) ∝ *k*^−*γ*^, where *γ* = 1 + 1/*ξ* [23, 24] and *P*(*k*) indicates the degree distribution. When generating SF networks with *γ* < 3, *i*_0_ is chosen appropriately to satisfy that the maximum degree $k_{\text{max}}<\sqrt{\langle k\rangle N}$; on the other hand, *i*_0_ = 1 when *γ* ≥ 3. When *i*_0_ = 1, the CL model is equivalent to the Goh—Kahng—Kim (GKK) model [25]. In this study, we avoided the emergence of self-loops and multiple edges. Specifically, SF networks were generated using the *static.power.law.game* function in the *igraph* package (version 0.7.1) of the R software (version 3.1.1; http://www.r-project.org). In the case of *γ* = ∞, we also considered the Erdős—Rényi (ER) random networks [1], in which the node degree follows a Poisson distribution whose mean is 〈*k*〉.

A simulated annealing (SA) method [8] was used to adjust the degree-degree correlation, measured using the assortative coefficient *r*_*a*_ [4] (see also S1 File). In particular, we minimized an energy $E=|r_{a}-r_{a}^{\text{obj}}|$, where $r_{a}^{\text{obj}}$ is the objective *r*_*a*_, through network modifications. In this SA method, a network is modified using degree-preserving rewiring [26], in which two randomly selected edges are rewired until 1% of the edges are rewired. For example, we consider two edges, A—B and C—D, where the letters and lines are the nodes and edges, respectively. Through this edge-rewiring algorithm, the edges A—D and C—B are obtained (see Ref [26] for details). A modified network, obtained as above, is accepted with the probability *p* = min[1, exp(*E*_0_ − *E*_1_)/*T*], where *E*_0_ and *E*_1_ are the energies obtained from the current network and the modified network, respectively, and *T* is the temperature. We started with *T* = 1000 and reduced *T* by updating as follows: *T* ← 0.995 × *T*. The above procedures were continued until *E* < 0.001 or *T* < 10^−16^.

Finding an MDS is NP-hard [21]; in particular, although it is considered as a binary integer programming problem, it is time consuming. Thus, we searched for an MDS in a network using linear programming relaxation as a binary integer programming problem, although it is possible to compute an MDS for moderate-sized networks [10, 11, 27]. We have confirmed that this relaxation does not affect the conclusions. In this study, the *lp* function in the R package *lpSolve* (version 5.6.10), a linear programming solver, was used. The size of an MDS was averaged over 100 realizations.

## Results

### Theoretical results

We analytically investigated the effect of degree-degree correlation (i.e., *r*_*a*_) on Γ, where Γ is the MDS size. However, it was difficult to mathematically reveal the relationship between Γ and arbitrary *r*_*a*_. Thus, we only evaluated the Γ of maximally assortative (respectively, maximally disassortative) networks, in which the exchange of any pair of edges does not increase (respectively, decrease) *r*_*a*_ (see “Assortative coefficient” in the S1 File).

#### The case of maximally assortative networks

Since a maximally assortative network has connections between nodes with similar degrees, it is considered as a collection of *k*-regular random graphs (*k* = *k*_min_, …, *k*_max_, where *k*_min_ and *k*_max_ are the minimum and maximum degrees, respectively, excluding degree 0 nodes). Let *n*_*k*_ be the number of nodes with degree *k*. Since the size of the MDS, Γ, of a *k*-regular random graph is approximately *n*_*k*_/(*k* + 1) (see “MDS in a *k*-regular random graph” in the S1 File), the Γ of a maximally assortative network (MAN) is derived as

$$\begin{array}{c} \Gamma_{\text{MAN}}=\sum_{k=0}^{k_{\max}}\frac{n_{k}}{k+1}. \end{array}$$ (1)

**Fig 1:**
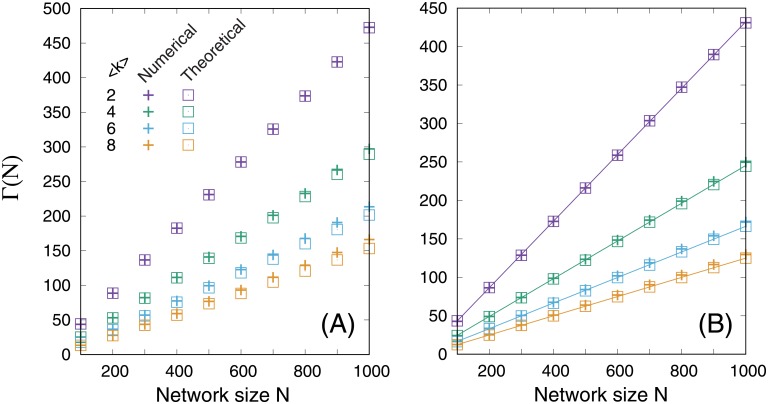
Network size dependency of the size of an MDS, Γ(*N*), in maximally assortative networks. (A) SF networks with *γ* = 2.5. (B) ER random networks. The cross symbols denote the numerical results. The open squares and solid lines are the theoretical estimations; in particular, they were obtained using Eqs (1) and (2), respectively.

Eq (1) is in excellent agreement with the numerical results (Fig 1). Maximally assortative networks were obtained using the SA method by setting $r_{a}^{\text{obj}}=1$, according to the CL and ER model networks. Γ ∝ *N* and the increase rate (slope) decreases with 〈*k*〉. This fact can be expected from the size of the MDS, $\Gamma_{\text{MAN}}^{\text{ERRG}}$, in a maximally assortative ER random graph (ERRG). $\Gamma_{\text{MAN}}^{\text{ERRG}}$ is approximately described as

$$\begin{array}{c} \Gamma_{\text{MAN}}^{\text{ERRG}}=\frac{N}{\left\langle k\right\rangle }\left(1-e^{-\langle k\rangle }\right) \end{array}$$ (2)

when 〈*k*〉/[*N* − 1] < 0.5 and *N* ≫ 0 (see “Size of an MDS in a maximally assortative Erdős—Rényi random graph” in the S1 File). The theoretical prediction based on Eq (2) is in excellent agreement with numerical results (Fig 1B); in addition to this, there is little difference in prediction accuracy between Eqs (1) and (2).

#### The case of maximally disassortative networks

In light of the characteristics of disassortativity (i.e., higher-degree nodes connect to lower-degree nodes), a maximally disassortative network is considered as a collection of regular random bipartite graphs (see “Maximally disassortative networks” in the S1 File). In particular, when considering an (*h*, *k*)-regular random bipartite graph (*h* > *k*) *G*(*V*_1_, *V*_2_, *E*), where each node in *V*_1_ has degree *h*, each node in *V*_2_ has degree *k*, and each edge connects a node in *V*_1_ and a node in *V*_2_, the maximally disassortative network is represented as

$$\begin{array}{c} B_{h_{1},k_{1}}\cup B_{h_{2},k_{2}}\cup \cdots \cup B_{h_{l},k_{l}}, \end{array}$$ (3)

where *B*_*h*_*i*_, *k*_*i*__ is an (*h*_*i*_, *k*_*i*_)-regular bipartite graph with *M*_*h*_*i*__ top nodes with degree *h*_*i*_ and *N*_*k*_*i*__ bottom nodes with degree *k*_*i*_. Note that *h*_1_ ≥ *h*_2_ ≥ … ≥ *h*_*l*_ and *k*_1_ ≤ *k*_2_ ≤ … ≤ *k*_*l*_. Actual structures will deviate from this form to some extent.

In short, the Γ of an (*h*, *k*)-regular random bipartite graph is useful for estimating the Γ of a maximally disassortative network. Let *α* = *m*/*n*, where *m* = |*V*_1_| and *n* = |*V*_2_|; an upper bound of the Γ of an (*h*, *k*)-regular random bipartite graph is approximately estimated as (see “MDS in a regular random bipartite graph” in the S1 File)

$$\begin{array}{c} f\left(\beta \right)=\left(\frac{\alpha }{1+k/(\alpha \beta )}+\frac{1}{1+k\beta }\right)n, \end{array}$$ (4)

where *β* is the ratio of the proportion of randomly selected nodes to the current top nodes to the proportion of randomly selected nodes to the current bottom nodes at each iteration in the recursive application of a probabilistic method (see “Size of an MDS in a maximally disassortative network” in the S1 File). The Γ of a regular random bipartite graph is obtained by minimizing Eq (4):

$$\begin{array}{c} \Gamma_{\text{RRGB}}=g\left(\alpha ,k,n\right)=\min_{\beta }f\left(\beta \right)=f\left(\beta_{0}\right). \end{array}$$ (5)

The size of a maximally disassortative network is estimated by summing up *g*(*α*, *k*, *n*) for all decomposed regular random bipartite graphs. Since *n*_*k*_ ≫ *n*_*k*′_ is expected for *k* ≪ *k*′, nodes (bottom nodes) with degree *k* may correspond to nodes (top nodes) with several degrees in each bipartite graph. We use *m*(*k*) to denote the number of the top nodes corresponding to the bottom nodes with degree *k*, where the definition of *m*(*k*) is given in the subsection “Size of an MDS in a maximally disassortative network” in the S1 File. Using this *m*(*k*), we redefine *α* by *α*(*k*) = *m*(*k*)/*n*_*k*_.

We also need to add some other factors: nodes with degree 0, and other nodes not counted by regular random bipartite graphs. To this end, we define *H* and *K* by $H=\max\{h|\sum_{d=k_{\min}}^{h}dn_{d}\leq \sum_{d=h+1}^{k_{\max}}dn_{d}\}$ and $K=\max\{k|\sum_{d=k_{\min}}^{H}dn_{d}\leq \sum_{d=k}^{k_{\max}}dn_{d}\}$, respectively. Then, the size of a maximally disassortative network (MDN) is estimated as

$$\begin{array}{c} \Gamma_{\text{MDN}}=n_{0}\;+\;\sum_{k=k_{\min}}^{H}g\left(\alpha \left(k\right),k,n_{k}\right)\;+\;\sum_{k=H+1}^{K}\frac{n_{k}}{k+1}, \end{array}$$ (6)

where we let *g*(*α*(*k*), *k*, *n*_*k*_) = *m*(*k*) if *k* = 1 and *g*(*α*, *k*, *n*) = 0 if *α* = 0 or *n* = 0. The third term reflects the effect of the nodes (with degree >0) not included in regular random bipartite graphs (“Size of an MDS in a maximally disassortative network” in the S1 File).

**Fig 2:**
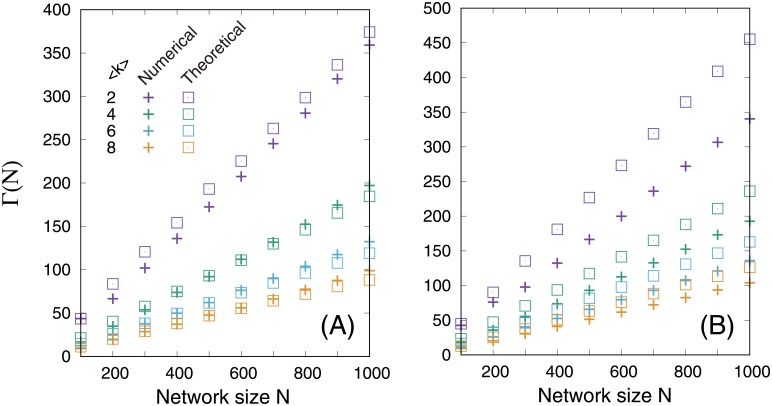
Network size dependency of the size of an MDS, Γ(*N*), in maximally disassortative networks. (A) SF networks with *γ* = 2.5. (B) ER random networks. The theoretical estimations (open squares) were obtained using Eq (6).

Eq (6) is in good agreement with the numerical results (Fig 2). As expected from the analysis [e.g., Eq (4)], Γ ∝ *N* and the increase rate (slope) declines with 〈*k*〉. However, the difference between numerical and estimated values becomes large in maximally disassortative networks of ER random networks and dense SF networks. This is because of the assumption of the theoretical estimation: we assumed that disassortative networks are completely decomposed into a collection of (*h*, *k*)-regular bipartite graphs and that the intermediate network structures of an (*h*, *k*)-regular graph are also regular, which are not necessarily true. Maximally disassortative networks were obtained using the SA method by setting $r_{a}^{\text{obj}}=-1$, according to the CL model networks and ER random networks; thus, the networks may be almost maximally disassortative. This fact may cause the difference between actual and estimated values.

### Numerical simulation

**Fig 3:**
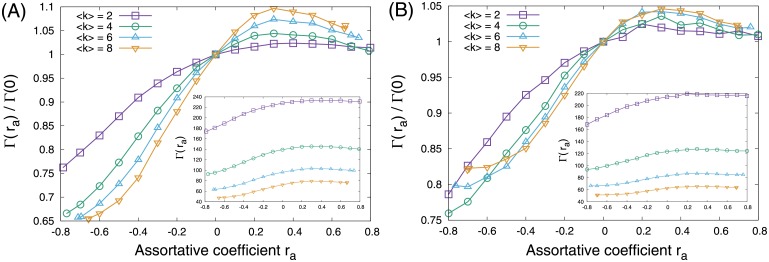
Relationship between the size of an MDS, Γ(*r*_*a*_), and assortative coefficient *r*_*a*_ in networks with *N* = 500. (A) Scale-free networks with *γ* = 2.5. (B) ER random networks. Note that the vertical axes of the main figure and the inset indicate relative Γ(*r*_*a*_)/Γ(0) and Γ(*r*_*a*_), respectively.

We performed numerical simulations to investigate the relationship between Γ and arbitrary *r*_*a*_, and found that Γ declines with decreasing *r*_*a*_ (Fig 3), whereas it is almost independent from *r*_*a*_ when *r*_*s*_ > 0. Similar results were observed in both SF networks and ER random networks; however, the effect of (dis)assortativity on Γ in SF networks is more significant than that in ER random networks.

The observed decrease in Γ with a negative *r*_*a*_ is due to the repulsion between high-degree nodes (i.e., hubs), which indicates disassortativity. Networks with higher-degree nodes (i.e., more heterogeneous networks) show smaller Γ [10, 11] because the trivial upper bound of Γ is described as *N* − *k*_max_, where *k*_max_ is the highest degree in a network. In a heterogeneous network (e.g., SF network), Γ becomes smaller when hubs are separated (i.e., high-degree nodes tend to connect to low-degree nodes) because hubs independently dominate many of the other (low-degree) nodes. In assortative networks, on the other hand, since high-degree nodes tend to connect to high-degree nodes, one hub dominates the other hubs. Thus, Γ hardly decreases because many low-degree nodes need to dominate the other nodes.

**Fig 4:**
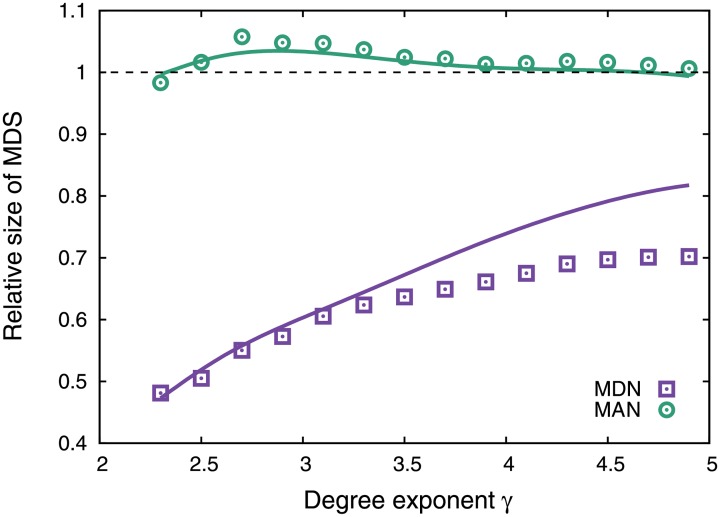
Degree exponent dependency of the difference between Γ_MAN_ and Γ_MDN_ in SF networks with *N* = 500 and 〈*k*〉 = 4. The symbols denote the numerical results. The solid lines are the theoretical predictions of Γ_MAN_ (i.e., Eq (1)) and Γ_MDN_ (i.e., Eq (6)). Γ_MAN_ (Γ_MDN_) is normalized by the size of the MDS in the uncorrelated network (i.e., network with *r*_*a*_ = 0).

These results indicate that the degree exponent *γ* (or degree heterogeneity) influences the difference between Γ_MAN_ and Γ_MDN_; in particular, they predict that more heterogeneous networks have smaller Γ_MDN_ because of the above reasons, whereas Γ_MAN_ is almost independent of *γ*. In fact, the difference becomes larger when *γ* is smaller (i.e., the degree heterogeneity becomes larger) (Fig 4). The accuracy of our theoretical estimation for Γ_MDN_ (i.e., Eq (6)) decreases with increasing *γ*. We discuss the reason in *Discussion and conclusions*.

Except for degree distributions, the SA method does not preserve the structural properties in networks; thus, it remains possible that the other properties mainly determine Γ. For example, clustering and modularity, which suggest community structure in complex networks [28], also influence system dynamics (e.g., epidemic dynamics [29]). To avoid this possibility, Pósfai et al. [8] evaluated these network parameters when investigating the effect of assortativity on maximum matching-based network controllability. Thus, we also investigated the relationship of Γ with clustering and modularity.

The clustering coefficient *C* is defined as the average ratio of the number of edges among the neighbors to the number of all possible connections among the neighbors [1]. The network modularity measure *Q* is defined as the fraction of edges that lie within modules rather than between modules, relative to that expected by chance [30]. We computed *C* and *Q* according to Refs. [1] and [30], respectively. In particular, the functions *transitivity* and *leading.eigenvector.community* in the R package *igraph* (version 0.7.1) were used for calculating *C* and *Q*, respectively.

**Fig 5:**
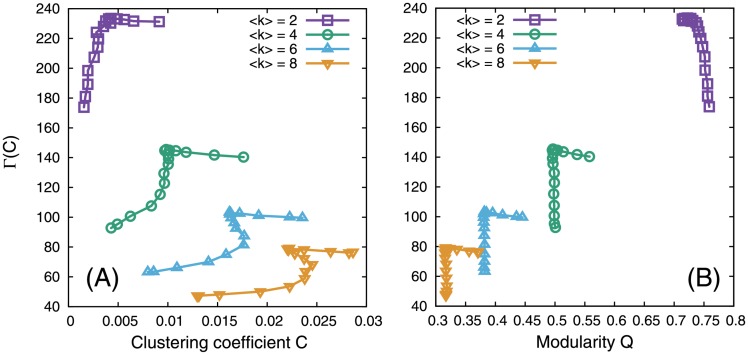
Correlation of the size of MDSs with the clustering coefficient (A) and modularity (B) in SF networks with *N* = 500 and *γ* = 2.5.

No clear relationship of Γ with *C* and *Q* was observed (Fig 5). This result indicates that *r*_*a*_ is the main factor in determining Γ.

## Discussion and Conclusions

In summary, we showed that disassortativity reduces the size of an MDS. This finding implies that disassortativity enhances network controllability. Whalen et al. [31] showed that the presence of symmetry in a network can decrease controllability, using small nonlinear systems. Our result strongly supports their claim because assortativity can be considered as a definition of symmetry; in particular, it suggests that the impact of disassortativity on network controllability can be generally concluded. Moreover, the results suggest that biological networks are relatively easy to control, compared with social networks, because biological networks (e.g., protein interaction networks) are disassortative, whereas social networks are assortative [4]. Liu et al. [3] suggested the difficulty in controlling social networks because it is necessary to control most individuals separately to control the whole system. Their observation and suggestion may be consistent with our theoretical predictions.

This result does not contradict a previous study [8], which showed that (dis-)assortativity does not increase network controllability in terms of structural controllability [3] because the model of control adopted in the MDS-based approach is different from the standard one. A relationship between an MDS and structural controllability is discussed in Ref [10], which states that, if every edge in a network is bi-directional and every node in an MDS can control all of its outgoing edge values independently, then the network is structurally controllable under a linear dynamic model. It should be noted that this MDS-based model employs a much stronger assumption than that used by the standard structural controllability model [3] in which only the values of driver nodes can be controlled. Thus, the MDS-based model may not be directly applicable to the control of typical dynamic systems. However, as mentioned in the *Introduction*, the MDS is a well-known concept in graph theory and has been applied to the design and control of various engineering systems. In addition, it is reported in several studies that nodes in an MDS and its variants tend to have important biological roles [9, 14–16]. For example, Wuchty [14] showed that proteins in an MDS are enriched with cancer-related and virus-targeted genes in protein interaction networks; furthermore, they found that such proteins have a higher impact on network resilience.

However, more careful examinations may be required to conclude the effect of degree-degree correlation on the size of an MDS. Specifically, we may need to consider the effect of other network properties on the size of an MDS. For example, Bianchin et al. [32] showed the importance of diameter in the controllability of complex networks, although the definition of network controllability in Ref [32] is different from that in our study. Disassortative networks may show a larger diameter than random networks do because of the repulsion between high-degree nodes.

Recent studies pointed out that the minimum number of driver nodes is not necessarily appropriate as a measure of the degree of controllability because the use of a few driver nodes may need a forbiddingly high amount of energy [32–36]. Instead, these studies focus on the energy needed to control network systems. In general, the minimum and maximum energies can be determined by evaluating the eigenvalues of the Gramian matrix: the minimum and maximum normalized energy costs are given by the reciprocal of the maximum and minimum eigenvalues [33]. By analyzing the Gramian matrix, researchers were able to perform various studies on the control energy, which include scaling laws with respect to control time [33], trade-off between the control energy and the number of control nodes [34], relationships between the control energy and the network diameter [32], algorithms for selecting a set of minimal control nodes under a given energy constraint [35], and relationships between the control energy and the number of control nodes in scale-free networks [36].

In particular, Yan et al. showed that, for a linear and continuous network control model with the power-law degree distribution *P*(*k*) ∝ *k*^−*γ*^ and self-loops, the maximum control energy scales sublinearly as *N*^1/(*γ*−1)^ when *N*_*D*_ = *N*, whereas it scales exponentially as *e*^*N*^ when *N*_*D*_ = 1, where *N* and *N*_*D*_ denote the number of network nodes and driver nodes, respectively [36]. In the MDS-based control model, it is assumed that each node in an MDS can control itself and its connecting edges independently [10]. On the other hand, by the definition of the MDS, each node in the network must be in an MDS or have at least one edge connecting to a node in the MDS. Therefore, it is meant that each node has an independent control input (if multiple control inputs are assigned to a node, we can use only the one that gives 0 values to the other inputs). This corresponds to the case of *N*_*D*_ = *N* in Ref [36], which suggests that the MDS-based control model requires relatively low energy costs, although it must control edges connecting to nodes in an MDS independently.

We proposed a method for estimating the size of an MDS in maximally (dis-)assortative networks from degree distributions. Although the estimation of MDS size or domination number is a central topic in graph theory, it has been limited in the context of random graphs without degree-degree correlations such as ER random networks [37], *k*-regular random graphs [38], and Barabási-Albert networks [39]. Our analytical approach is applicable to the estimation of the domination number of a network with a given degree distribution. However, our theoretical estimations (i.e., Eqs (1) and (6)) have limitations. In particular, the estimation in ER random networks is better than that in SF networks in the maximally assortative case; on the other hand, the opposite tendency was observed in the maximally disassortative case. This is because of the difference in degree heterogeneity between ER random networks and SF networks. The theoretical estimation in the maximally assortative case (i.e., Eq (1)) assumes a collection of *k*-regular random graphs. The assumption of *k*-regular random graphs is appropriate in ER random networks because node degree is almost similar. For a large *k*, however, this assumption is hardly satisfied in SF networks because of the existence of a few high-degree nodes (i.e., hubs) due to heterogeneous (power-law) degree distributions. Thus, Eq (1) shows a lower prediction accuracy in SF networks. On the other hand, the theoretical estimation in the maximally disassortative case (i.e., Eq (6)) considers the assumption of a group of (*h*, *k*)-regular random bipartite graphs. This assumption is not appropriate in ER random networks when the difference between *h* and *k* is large (i.e., the maximally disassortative case), although it is relatively suitable in SF networks because of the hubs. In particular, it is difficult to obtain (*h*, *k*)-regular random bipartite graphs in such a case (i.e., *h* ≫ *k*) because node degree is almost similar in ER random networks; thus, Eq (6) exhibits a lower accuracy.

In this study, we focused on the MDS in maximally assortative and disassortative networks. However, the proposed methodology may be extended to the general degree-degree correlation case by making use of a weighted mixture of (*h*, *k*)-regular bipartite networks. The proposed methodology (i.e., combination of network decomposition and recursive application of a probabilistic method) may also be applied to the analysis of other graph theoretic properties (e.g., maximum matching, independent set) in assortative/disassortative networks. Our study could lead to a deeper understanding of network controllability and other properties of complex networks.

## Supporting Information

S1 File Supplementary information. (PDF)
