# Supplementary material for: Analysis of the Effect of Degree Correlation on the Size of Minimum Dominating Sets in Complex Networks
Source: PLoS One. 2016 Jun 21;11(6):e0157868. doi: 10.1371/journal.pone.0157868 (PMC4915616; doi:10.1371/journal.pone.0157868)
Supplement: S1 File — (PDF) [file pone.0157868.s001.pdf]

Supplementary Information for "Analysis of the Effect of Degree Correlation on the Size of Minimum Dominating Sets in Complex Networks"

Kazuhiro Takemoto and Tatsuya Akutsu

Preliminary

Let $G(V, E)$ denote an undirected graph with a set of nodes V and a set of edges E. We do not allow self-loops but allow multi-edges in the theoretical analysis (we will discuss appropriateness on the assumption of multi-edges later). In the following, N and M denote the number of nodes and edges in a given graph, respectively (i.e., $N = |V|$ and $M = |E|$), unless otherwise stated. A subset U of V is called a *dominating set* if for all $v \in V$, $v \in U$ holds or v has a neighbor $u \in U$ (i.e., there exists u such that $\{v, u\} \in E$ and $u \in U$). A dominating set with the minimum number of nodes is called a *minimum dominating set*. For a node v, $d(v)$ denotes the degree of v, the number of edges connecting to v. In this paper, we mainly consider scale-free networks in which the degree distribution $P(k)$ ($P(k)$ denotes the probability that a given node has degree k) follows a power-law, i.e., $P(k) = c_0 k^{-\gamma}$ holds for $k = 1, \cdots, k_{max}$ where $k_{\max}$ denotes the maximum degree, γ is a positive constant depending on the network, and c_0 is a normalization constant so that $\sum_{k=1}^{k_{\max}} P(k) = 1$ holds. It is to be noted that we usually consider the average case behavior or property in the analysis of SF networks because scale-free networks are a special type of random graphs. Since it is quite difficult in many cases to analyze the average case behavior exactly, approximate analysis methods such as mean-field approximation are often employed in studies on SF networks.

Assortative coefficient

Recall that the *assortative coefficient* r_a is given by

$$r_a = \frac{\left(\frac{1}{M}\sum_{e \in E} e_1 e_2\right) - \left(\frac{1}{M}\sum_{e \in E} \frac{1}{2}(e_1 + e_2)\right)^2}{\left(\frac{1}{M}\sum_{e \in E} e_1^2 + e_2^2\right) - \left(\frac{1}{M}\sum_{e \in E} \frac{1}{2}(e_1 + e_2)\right)^2}, \quad (1)$$

where e_1 and e_2 denote the degrees of the end points of an edge e [1].

A graph is called *maximally assortative* (respectively, *maximally disassortative*) if the exchange of any pair of edges does not increase (respectively, decrease) r_a.

We here characterize maximally assortative and maximally disassortative networks.

In a maximally disassortative (respectively, maximally assortative) network, $d(v_i) \geq d(v_l) \geq d(v_j) \geq d(v_k)$ or $d(v_l) \geq d(v_i) \geq d(v_k) \geq d(v_j)$ (respectively, $d(v_k) \geq d(v_l)$ or $d(v_j) \geq d(v_i)$) holds for any pair of edges $\{v_i, v_k\}$ and $\{v_j, v_l\}$, where $d(v_i) \geq d(v_k)$ and $d(v_l) \geq d(v_j)$.

First, we consider the disassortative case. We show the case of $d(v_i) > d(v_l)$. The case of $d(v_i) < d(v_l)$ can be explained in an analogous way, and the condition is always satisfied if $d(v_i) = d(v_l)$.

Suppose that there exists a pair of edges not satisfying the above condition. Then, $d(v_k) > d(v_j)$ should hold.

Let $d(v_l) = x$, $d(v_k) = x + b$, $d(v_i) = x + b + c$, and $d(v_j) = x - a$ (see Fig 1). Then, $a \geq 0$ and $c \geq 0$ clearly hold, and $b > 0$ if and only if $d(v_k) > d(v_l)$. Furthermore, $b + c > 0$ holds from $d(v_i) > d(v_l)$, and $a + b > 0$ holds from $d(v_k) > d(v_j)$.

Here, we remove these two edges and, instead, add two edges: $\{v_i, v_j\}$ and $\{v_k, v_l\}$. It is easy to verify that the assortative coefficient is affected only for the part of $\sum_{e \in E} e_1 e_2$ by this exchange. Let Δ_d denote the change of this value.

Then, we have

$$\begin{aligned}
\Delta_d &= (d(v_i)d(v_j) + d(v_k)d(v_l)) - (d(v_i)d(v_k) + d(v_j)d(v_l)) \\
&= ((x+b+c)(x-a) + (x+b)x) - ((x+b+c)(x+b) + (x-a)x) \\
&= -(a+b)(b+c) \\
&< 0. \qquad (2)
\end{aligned}$$

Thus, r_a decreases by this exchange.

Next, we consider the assortative case. We show the case of $d(v_i) > d(v_l)$. The cases of $d(v_i) = d(v_l)$ and $d(v_i) < d(v_l)$ can be explained in similar ways. Suppose that there exists a pair of edges not satisfying the above condition. Then, $d(v_l) > d(v_k)$ should hold.

As in the above, we let $d(v_l) = x$, $d(v_k) = x + b$, $d(v_i) = x + b + c$, and $d(v_j) = x - a$. Then, $a \geq 0$ and $c \geq 0$ clearly hold, $b < 0$ holds from $d(v_l) > d(v_k)$, and $b + c > 0$ holds from $d(v_i) > d(v_l)$.

Here, we remove two original edges and, instead, add two edges: $\{v_i, v_l\}$ and $\{v_k, v_j\}$. Let Δ_a denote the change of $\sum_{e \in E} e_1 e_2$ by this exchange.

Then, we have

$$\begin{aligned}
\Delta_a &= (d(v_i)d(v_l) + d(v_k)d(v_j)) - (d(v_i)d(v_k) + d(v_j)d(v_l)) \\
&= ((x+b+c)x + (x+b)(x-a)) - ((x+b+c)(x+b) + (x-a)x) \\
&= -b(a+b+c) \\
&> 0. \qquad (3)
\end{aligned}$$

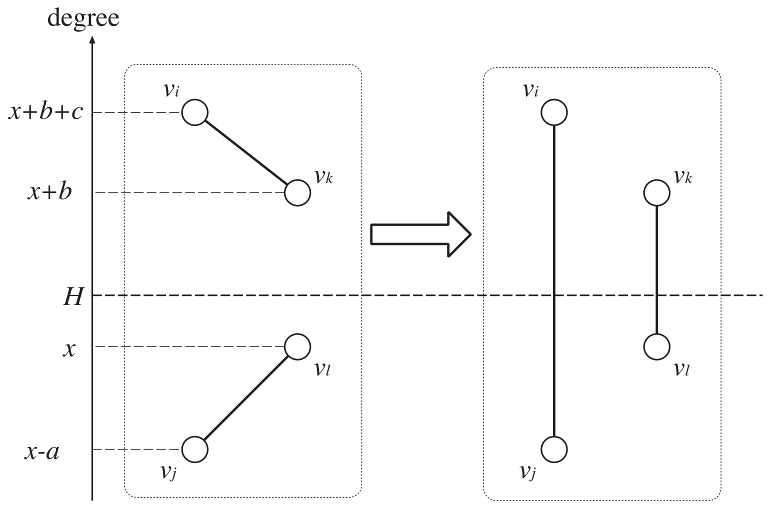

Figure 1: Schematic diagram of edge switching.

Thus, r_a increases by this exchange.

It is to be noted that multi-edges may be generated by the exchanges mentioned in the above proof. Although our numerical simulations avoid the emergence of multi-edges, the assumption that the generation of multi-edges is allowed poses few problems. In particular, we numerically confirmed that few multi-edges were generated through this edge exchange, using a method for rewiring edges based on node degrees [2, 3] that is essentially similar to our approach (Fig 1). (a) Two edges are randomly selected, and the four nodes are ranked in order of their degrees. (b) The highest degree node is connected to the lowest degree node. The other edge is drawn between the two remaining nodes. Steps (a) and (b) are repeated until the rewiring of all edges is completed. After generating random networks with $\langle k \rangle = 8$ (i.e., dense networks) using the CL model and ER model, we obtained disassortative networks using the above method. The number of multi-edges was averaged over 100 realizations. We found that multi-edges of <4% emerged when $N = 100$ in both cases (i.e., CL model and ER model). Moreover, multi-edges of <1% were generated when $N \geq 500$ in both cases.

The case of maximally assortative networks

It can be seen from the above result that a maximally assortative network has connections only between nodes with similar degrees. If there are many nodes at each degree, it is a collection of k-regular random graphs with a small number of edges between degrees k and $k + 1$ for each k. Therefore, the structure of a maximally assortative network is approximated by a collection of k-regular random graphs ($k = k_{\min}, \ldots, k_{\max}$, where $k_{\min}$

and $k_{\max}$ are the minimum and maximum degrees, respectively, excluding degree 0 nodes).

MDS in a k-regular random graph

On the basis of the above discussion, we consider the MDS of a k-regular random graph. Let $G(V, E)$ be a k-regular random graph, where $|V| = n$. For $V' \subseteq V$, $N(V')$ denotes the set of nodes that are neighbors of V' (i.e., $N(V') = \{u|\{u, v\} \in E, v \in V'\}$). To estimate an upper bound of the size of an MDS for $G(V, E)$, we construct a DS as follows, where β is a very small constant as explained later:

(i) Let $G_1(V_1, E_1) = G(V, E)$, $DS \leftarrow \{\}$, and $i \leftarrow 1$.

(ii) Randomly choose a set W_i of $\lceil \beta |V_i| \rceil$ nodes and delete nodes in $W_i \cup N(W_i)$.

(iii) Let the resulting graph be $G_{i+1}(V_{i+1}, E_{i+1})$, $DS \leftarrow DS \cup W_i$, and $i \leftarrow i + 1$.

(iv) Repeat (ii)-(iii) until G_i becomes empty.

(v) $DS \leftarrow DS \cup V_i$.

In the following analysis, we ignore the size of V_i added in step (v) because it is small.

Here, we estimate the expected size of DS. We begin with the first iteration step (i.e., $i = 1$). The number of nodes not belonging to or dominated by W_1 (i.e., the size of $V_1 - (W_1 \cup N(W_1)))$ is estimated as

$$(n - \beta n) \cdot \left(1 - \frac{\beta n}{n}\right)^k = n(1 - \beta)^{k+1}$$

since each node (with degree k) in $V_1 - W_1$ does not connect to (i.e., it is not dominated by) any node in W_1 with probability $(1 - \beta n/n)^k$. Therefore, the number of nodes in G_2 is estimated as $n(1 - \beta)^{k+1}$.

G_2 is not necessarily a k-regular graph because the edges between $N(W_1)$ and $V_1 - (W_1 \cup N(W_1))$ had been removed. However, we treat G_2 as if it were a k-regular random graph because the number of such removed edges is considered to be much smaller than the number of edges in G_2. Let $n_2 = n(1 - \beta)^{k+1}$, which is the estimated number of nodes in G_2. Then, the number of nodes in G_2 that are not belonging to or dominated by W_2 is estimated as

$$(n_2 - \beta n_2) \cdot \left(1 - \frac{\beta n_2}{n_2}\right)^k = n_2(1 - \beta)^{k+1} = n(1 - \beta)^{2(k+1)}.$$

By repeating this procedure, we can estimate the number of nodes n_i in G_i as

$$n_i = n(1 - \beta)^{(i-1)(k+1)}. \tag{4}$$

Since the size f of the above constructed DS is given by

$$W_1 + W_2 + \cdots + W_i + \cdots = \beta n_1 + \beta n_2 + \cdots + \beta n_i + \cdots,$$

f is estimated as

$$f = \beta n(1 + (1-\beta)^{k+1} + (1-\beta)^{2(k+1)} + \cdots) = \frac{\beta n}{1-(1-\beta)^{k+1}}. \tag{5}$$

By using $(1-\beta)^{k+1} \approx 1 - \beta(k+1)$ for very small β, we can simplify the estimated size to

$$\frac{\beta n}{1-(1-\beta(k+1))} = \frac{n}{k+1}. \tag{6}$$

Since the size of an MDS in a k-regular graph is lower bounded by $n/(k+1)$, the size of an MDS in a k-regular random graph is estimated as $n/(k+1)$.

MDS in a maximally assortative network

As mentioned above, a maximally assortative network is considered as a collection of k-regular random graphs; thus, using Eq. (6), we can derive its Γ as

$$\Gamma_{\text{MAN}} = \sum_{k=0}^{k_{\max}} \frac{n_k}{k+1}, \tag{7}$$

where n_k denotes the number of nodes with degree k.

Size of an MDS in a maximally assortative Erdős–Rényi random graph

$\Gamma_{\text{MAN}}^{\text{ERRG}}$ is approximately described as follows. The degree distribution $P(k)$ of an ER random graph is known to be a binomial distribution [4]:

$$P(k) = \binom{N-1}{k} p^k (1-p)^{N-1-k},$$

where $p = \langle k\rangle/(N-1)$ [4].

Since $n_k = NP(k)$, $\Gamma_{\text{MAN}}^{\text{ERRG}}$ is estimated as

$$\Gamma_{\text{MAN}}^{\text{ERRG}} = \sum_{k=0}^{k_{\max}} \frac{NP(k)}{k+1} \approx \sum_{k=0}^{\infty} \frac{NP(k)}{k+1} = \frac{N-1}{\langle k\rangle}\left[1 - \left(1 - \frac{\langle k\rangle}{N-1}\right)^N\right] \tag{8}$$

when $\langle k\rangle/[N-1] < 0.5$.

The above equation is approximately described as

$$\Gamma_{\text{MAN}}^{\text{ERRG}} \approx \frac{N}{\langle k\rangle}\left(1 - \mathrm{e}^{-\langle k\rangle}\right) \tag{9}$$

because $(1 - a/(N-1))^N \approx \mathrm{e}^{-a}$ and $N - 1 \approx N$ when $N \gg 0$. From this equation, we can obtain a trivial result that the size of an MDS is equivalent to N when $\langle k\rangle \to 0$.

The case of maximally disassortative networks

Maximally disassortative networks

It can be seen from the result of the "assortative coefficient" that a maximally disassortative network G ideally has a structure that is a collection of (h,k)-regular random bipartite graphs ($h > k$) (see also Fig 2):

$$B_{h_1,k_1} \cup B_{h_2,k_2} \cup \cdots \cup B_{h_l,k_l}$$

where B_{h_i,k_i} is an (h_i,k_i)-regular bipartite graph with M_{h_i} top nodes with degree h_i and N_{k_i} bottom nodes with degree k_i. Of course, real structures will deviate from it to some extent, as shown by the dotted edges in Fig 2.

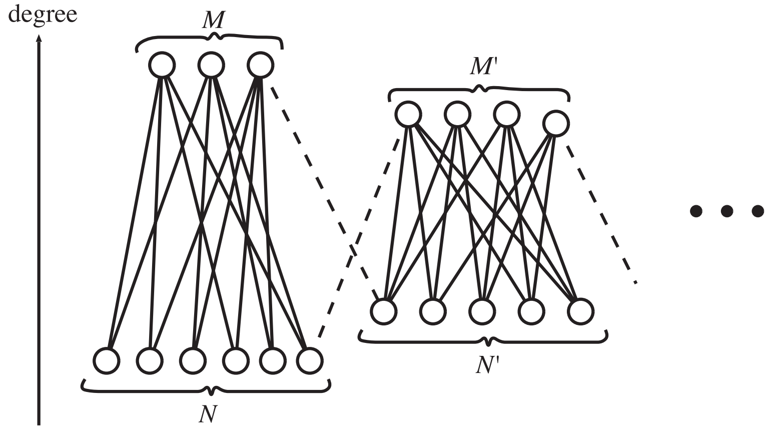

Figure 2: Structure of a maximally, but not ideally, disassortative network.

MDS in a regular random bipartite graph

On the basis of the above discussion, we consider an (h,k)-regular random bipartite graph $G(V_1,V_2,E)$ where each node in V_1 has degree h, each node in V_2 has degree k, and each edge connects a node in V_1 and a node in V_2. Let $m = |V_1|$ and $n = |V_2|$, from which $hm = kn$ follows.

To estimate the size of an MDS for $G(V_1,V_2,E)$, we consider a procedure (see also Fig 3) similar to that in the case of maximally assortative networks, where λ and μ are small constants.

(i) Let $G^1(V_1^1,V_2^1,E^1) = G(V_1,V_2,E)$, $DS \leftarrow \{\}$, and $i \leftarrow 1$.

(ii) Randomly choose a set W_i of $\lceil \lambda |V_1^i| \rceil$ nodes in V_1^i, delete nodes in $W_i \cup N(W_i)$, and let $V_3^i = V_2^i - N(W_i)$.

(iii) Randomly choose a set U_i of $\lceil \mu |V_3^i| \rceil$ nodes in V_3^i and delete nodes in $U_i \cup N(U_i)$.

(iv) Let $G^{i+1}(V_1^{i+1}, V_2^{i+1}, E^{i+1})$ be the resulting graph, $DS \leftarrow DS \cup W_i \cup U_i$, and $i \leftarrow i+1$.

(v) Repeat (ii)-(iv) until G^i becomes empty.

(vi) $DS \leftarrow DS \cup V_1^i \cup V_2^i$.

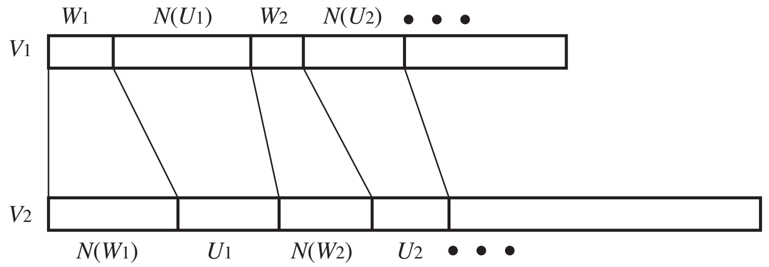

Figure 3: Illustration for W_i and U_i.

Here, we estimate the expected size of DS. Let $m_i = |V_1^i|$ and $n_i = |V_2^i|$. We begin with the first iteration step (i.e., $i = 1$). The size of W_1 is approximated as λm. After step (ii), the expected size of V_3^1 is estimated as

$$\left(1 - \frac{\lambda m}{m}\right)^k n = (1-\lambda)^k n.$$

Thus, the size of U_1 is estimated as

$$\mu(1-\lambda)^k n.$$

Therefore, after step (iii), the expected sizes of V_1^2 and V_2^2 are estimated as

$$(1-\lambda)m\left(1 - \frac{|U_1|}{|V_3^1|}\right)^h = (1-\lambda)(1-\mu)^h m$$

and

$$|V_3^1| - |U_1| = (1-\mu)(1-\lambda)^k n,$$

respectively.

Although G^2 is not necessarily an (h,k)-regular bipartite graph (because the edges between $N(W_1)$ and V_1 and those between $N(U_1)$ and V_2 had been removed), we treat G^2 (and G^i for $i > 2$) as if it were an (h,k)-regular random bipartite graph. Repeating the same argument as above for each i, we can estimate the sizes of W_i and U_i as

$$\lambda(1-\lambda)^{i-1}(1-\mu)^{h(i-1)} m$$

and

$$\mu(1-\mu)^{i-1}(1-\lambda)^{ki}n,$$

respectively.

Since the size f of the above constructed DS is given by

$$(W_1 + W_2 + \cdots + W_i + \cdots) + (U_1 + U_2 + \cdots + U_i + \cdots),$$

f is estimated as Eq. (10).

$$\begin{aligned}
f &= \lambda\Big(1 + (1-\lambda)(1-\mu)^h + (1-\lambda)^2(1-\mu)^{2h} + \cdots (1-\lambda)^{i-1}(1-\mu)^{h(i-1)} + \cdots\Big)m \\
&\quad + \mu(1-\lambda)^k\Big[1 + (1-\mu)(1-\lambda)^k + (1-\mu)^2(1-\lambda)^{2k} + \cdots \\
&\quad \cdots + (1-\mu)^{i-1}(1-\lambda)^{k(i-1)} + \cdots\Big]n \\
&= \left(\frac{\lambda}{1-(1-\lambda)(1-\mu)^h}\right)m + \mu(1-\lambda)^k\left(\frac{1}{1-(1-\mu)(1-\lambda)^k}\right)n. \qquad (10)
\end{aligned}$$

Here, we let $\alpha = m/n$ and $\beta = \lambda/\mu$. By using $(1-x)^k \approx 1-kx$ (for small x) and $h = k/\alpha$, we can approximate f as

$$\begin{aligned}
f &\approx \left(\frac{\alpha\lambda}{\lambda + h\mu - h\lambda\mu} + \frac{\mu(1-\lambda)^k}{\mu + k\lambda - k\lambda\mu}\right)n \\
&\approx \left(\frac{\alpha\lambda}{\lambda + h\mu} + \frac{\mu(1-\lambda)^k}{\mu + k\lambda}\right) \\
&\approx \left[\frac{\alpha}{1+\frac{k}{\alpha\beta}} + (1-\lambda)^k\left(\frac{1}{1+k\beta}\right)\right]n \qquad (11)
\end{aligned}$$

Approximating $(1-\lambda)^k$ by 1, we have the following simple form:

$$f(\beta) = \left(\frac{\alpha}{1+\frac{k}{\alpha\beta}} + \frac{1}{1+k\beta}\right)n. \qquad (12)$$

By solving $\mathrm{d}f(\beta)/\mathrm{d}\beta = 0$, we have β_0 minimizing Eq (12):

$$\beta_0 = \frac{-2k(1-\frac{1}{\alpha}) + \sqrt{4(\frac{k^2}{\alpha}-1)^2 - 3}}{2(k^2-1)}. \qquad (13)$$

Therefore, we get an estimate $g(\alpha, k, n)$ of the upper bound of the MDS size of an (h, k)-regular random bipartite graph $G(V_1, V_2, E)$ with $|V_2| = n$ as

$$g(\alpha, k, n) = \min_{\beta} f(\beta) = f(\beta_0). \qquad (14)$$

It is interesting to note that, if we let $\beta = 1$, Eq (12) will have the following form

$$\frac{m}{1+h} + \frac{n}{1+k}$$

It suggests that the expected size of an MDS for $G(V_1, V_2, E)$ is not greater than the sum of the expected sizes of MDSs for an h-regular random graph with m nodes and a k-regular random graph with n nodes.

Size of an MDS in a maximally disassortative network

By combining the MDSs for bipartite networks, we estimate an upper bound of the size of an MDS in a maximally disassortative network.

First, we consider the continuous case. We assume that the degree distribution follows a power-law $P(k) = ck^{-\gamma}$ beginning with the minimum degree $D = k_{min}$, where c is determined from $cN\int_D^{+\infty} k^{-\gamma}dk = N$ (we can replace $+\infty$ with N or such value as $\sqrt{N}$ without significantly affecting the results). Then, the average degree is given by $cN\int_D^{+\infty} k \cdot k^{-\gamma}dk = cN(\gamma-2)D^{2-\gamma}$.

According to the property of MDNs (see also S1 Appendix), we assume that the nodes with degree y are connected to the nodes with degree x, where y is determined from x as

$$y = \left(D^{2-\gamma} - x^{2-\gamma}\right)^{-\frac{1}{\gamma-2}}, \tag{15}$$

which comes from

$$cN\int_y^{+\infty} k \cdot k^{-\gamma} \;=\; cN\int_D^{x} k \cdot k^{-\gamma}.$$

It should be noted that $y = x$ holds when

$$x = 2^{\frac{1}{\gamma-2}} \cdot D. \tag{16}$$

Let $\overline{M} = cN\int_y^{+\infty} k^{-\gamma}dk$ and $\overline{N} = cN\int_D^{x} k^{-\gamma}dk$. Then, the ratio $\alpha(x)$ of the number of nodes with degree y to the number of nodes with degree x is given by

$$\begin{aligned}\alpha(x) &= \frac{d\overline{M}}{d\overline{N}} = \frac{d\overline{M}}{dx}\bigg/\frac{d\overline{N}}{dx} = \left(\frac{d\overline{M}}{dy}\cdot\frac{dy}{dx}\right)\bigg/\frac{d\overline{N}}{dx} \\ &= \left[(-y^{-\gamma})\cdot\left(-x^{1-\gamma}\cdot\left(D^{2-\gamma}-x^{2-\gamma}\right)^{-\frac{\gamma-1}{\gamma-2}}\right)\right]/x^{-\gamma} \\ &= x\cdot\left(D^{2-\gamma}-x^{2-\gamma}\right)^{\frac{1}{\gamma-2}}.\end{aligned} \tag{17}$$

By using Eq. (14), we can estimate the ratio $f(\gamma)$ of the size of an MDS to the number of nodes n as

$$f(\gamma) \;=\; \frac{\int_D^{2^{\frac{1}{\gamma-2}}\cdot D} g(\alpha(k), k, P(k)N)dk}{n\int_D^{+\infty} P(k)dk}. \tag{18}$$

Since real degree distributions significantly deviate from $cNk^{-\gamma}$, we also consider empirical distributions. Let $k_{\max}$ and $k_{\min}$ be the maximum and minimum degrees (except degree 0 nodes) in a given graph G, respectively. First, we define H by

$$H = \max\{h | \sum_{d=k_{\min}}^{h} dn_d \leq \sum_{d=h+1}^{k_{\max}} dn_d\},$$

where n_d is the number of nodes with degree d. It means that the node with degree at most H and the nodes with degree greater than H correspond to the bottom and top nodes, respectively. Since $n_k \gg n_{k'}$ is expected for $k \ll k'$, nodes (bottom nodes) with degree k may correspond to nodes (top nodes) with several degrees in each bipartite graph. We use $m(k)$ to denote the number of top nodes corresponding to the bottom nodes with degree k. To this end, we define functions $k_{\text{top}}(k)$ and $m(k)$ (see Fig 4) by

$$k_{\text{top}}(k) = \max\{k' | \sum_{d=k'}^{k_{\max}} d \cdot n_d \geq \sum_{d=k_{\min}}^{k} d \cdot n_d\} \tag{19}$$

and

$$\begin{aligned} m(k) \quad = \quad & \left(\sum_{d=k_{\text{top}}(k)-1}^{k_{\text{top}}(k-1)-1} n_d \right) + \frac{1}{k_{\text{top}}(k)} \left(\sum_{d=k_{\min}}^{k} dn_d - \sum_{d=k_{\text{top}}(k)-1}^{k_{\max}} dn_d \right) \\ & - \frac{1}{k_{\text{top}}(k-1)} \left(\sum_{d=k_{\min}}^{k-1} dn_d - \sum_{d=k_{\text{top}}(k-1)-1}^{k_{\max}} dn_d \right). \end{aligned} \tag{20}$$

Using this $m(k)$, we redefine α by $\alpha(k) = m(k)/n_k$.

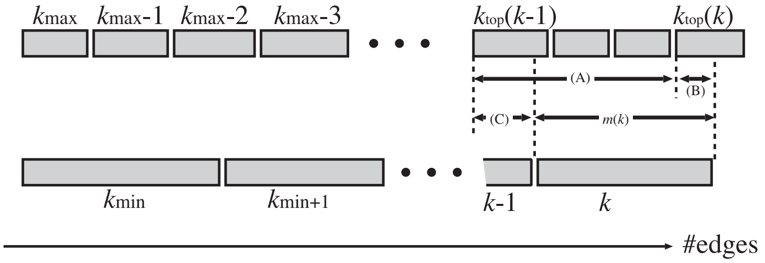

Figure 4: Illustration for $m(k)$. (A), (B), and (C) correspond to the first, second, and last terms in Eq (20), respectively, where these terms represent the numbers of nodes (not the numbers of edges).

In addition to the above factor, we need to add the contributions of nodes not included

in the bipartite subgraphs. We define K by

$$K = \max\{k | \sum_{d=k_{\min}}^{H} dn_d \leq \sum_{d=k}^{k_{\max}} dn_d\}. \quad (21)$$

Then, we add a factor of $\frac{n_k}{k+1}$ for a dominating set of a d-regular graph of each degree k between $H+1$ and K, where only a part of nodes with degree K, which do not correspond to degree H nodes, are counted as n_K (see Fig 5). It is also necessary to add the number of degree 0 nodes because they are always included in any MDS.

Summing up all the factors, we can estimate the size of an MDS by

$$\Gamma_{\text{MDN}} = n_0 + \sum_{k=k_{\min}}^{H} g(\alpha(k), k, n_k) + \sum_{k=H+1}^{K} \frac{n_k}{k+1}, \quad (22)$$

where we let $g(\alpha(k), k, n_k) = m(k)$ if $k = 1$ and $g(\alpha, k, n) = 0$ if $\alpha = 0$ or $n = 0$.

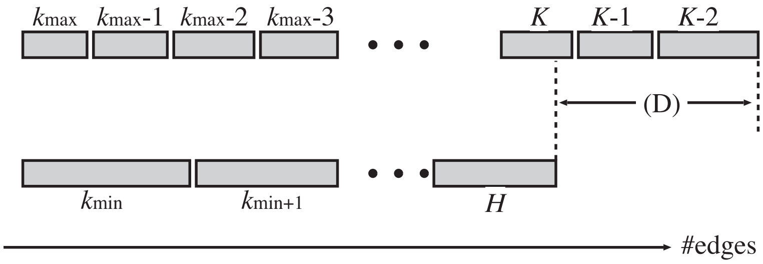

Figure 5: Illustration for H and K, where $K - 2 = H + 1$ in this case. MDSs for the nodes in (D) are taken into account.

References

[1] Newman MEJ, Assortative mixing in networks. Phys Rev Lett. 2002; 89(20): 208701.

[2] Trusina A, Maslov S, Minnhagen P, Sneppen K. Hierarchy measures in complex networks. Phys Rev Lett. 2004; 92(17): 178702.

[3] Takemoto K, Oosawa C. Introduction to complex networks: measures, statistical properties, and models. in: Dehmer M, Basak SC, editors. Statistical and Machine Learning Approaches for Network Analysis. New York: John Wiley & Sons; 2012. pp. 45–75.

[4] Albert R, Barabási A-L. Statistical mechanics of complex networks. Rev Mod Phys. 2002; 74(1): 47–97.
